# Analysis of online antenatal education class use via a mobile terminal app during the COVID-19 pandemic

**DOI:** 10.1186/s12884-022-04745-5

**Published:** 2022-05-16

**Authors:** Xiao-Wen Chen, Li-Yuan Jiang, Ya Chen, Li-Fang Guo, Xu-Hong Zhu

**Affiliations:** Department of Obstetrics and Gynecology, Hangzhou Women’s Hospital (Hangzhou Maternity and Child Health Care Hospital), No. 369, Kun Peng Road, Hangzhou, 310003 Zhejiang China

## Abstract

**Objective:**

To understand the use of online antenatal education classes accessed via the Mother and Child Health Handbook app during the COVID-19 pandemic in order to provide a basis and suggestions for optimizing Internet education during pregnancy under public health emergencies.

**Methods:**

We compared and analyzed the use of online antenatal education classes via the Mother and Child Health Handbook app in Hangzhou in 2019 and 2020 (during the COVID-19 pandemic).

**Results:**

Between January 1, 2019, and December 31, 2020, a total of 229,794 pregnant women created files and registered for the app, including 124,273 women in 2019 and 105,521 women in 2020. More pregnant women participated in online antenatal education learning (*n* = 36,379/34.5% vs. 29,226/23.5%, *p* = 0.000) in 2020 than in 2019. The proportion of pregnant women in the 18–34-year-old group who participated in online learning was higher than that in the advanced age group, and the difference was statistically significant (2019: 24.3% vs. 18.8%, *p* = 0.000) (2020: 35.7% vs. 27.4%, *p* = 0.000). More pregnant women accessed online antenatal education during early pregnancy (*n* = 13,463/37.0% vs. 9088/31.1%, *p* = 0.000) in 2020 than in 2019. Similar percentages of pregnant women participated in online antenatal education during mid-pregnancy (*n* = 15,426/52.8% vs. 19,269/53.0%, *p* = 0.639) in 2019 and 2020. Fewer pregnant women accessed online antenatal education during late pregnancy (*n* = 10,246/28.2% vs. 9476/32.4%, *p* = 0.000) in 2020 than in 2019. Fewer pregnant women choose to take 'Puerperal Health' courses in 2020 than in 2019 (early pregnancy: 36.20% vs. 42.79%, *p* = 0.000; mid-pregnancy: 41.65% vs. 48.19%, *p* = 0.000; late pregnancy: 55.31% vs. 58.41%, *p* = 0.000). Fewer pregnant women choose to take 'Psychological Adjustment' courses in 2020 than in 2019 (early pregnancy: 21.59% vs. 29.60%, *p* = 0.000; mid-pregnancy: 26.20% vs. 40.50%, *p* = 0.000; late pregnancy: 12.79% vs. 42.53%, *p* = 0.000). More pregnant women choose to study 'Nutrition and Exercise' in 2020 than in 2019 (early pregnancy: 44.48% vs. 25.95%, *p* = 0.000; mid-pregnancy: 47.77% vs. 40.75%, *p* = 0.000; late pregnancy: 55.94% vs. 42.99%, *p* = 0.000). “Pregnancy Care and Fetal Development” was the most selected course by pregnant women in early pregnancy (2019: 67.50%; 2020: 71.39%) and middle pregnancy (2019: 67.01%; 2020: 82.05%), and the proportion in 2020 was higher than it was in 2019. “Baby care” was the most selected course by pregnant women in late pregnancy, and the proportion in 2020 was higher than it was in 2019 (78.31% vs. 72.85%).

**Conclusion:**

During the COVID-19 pandemic, online antenatal education was well-used by pregnant women. More women participated in the online antenatal education modules during the COVID-19 pandemic than during 2019.The proportion of choosing different courses for pregnant women before and after the COVID-19 epidemic varied, and the learning course needs of pregnant women in different trimesters were different.

## Introduction

Traditional pregnancy health education in the Hangzhou region is mainly based on antenatal education classes in community hospitals and other medical institutions, face-to-face conversations between doctors and pregnant women during maternity checkups, supplemented by online self-learning using the Mother and Child Health Handbook app. Since the beginning of 2020, due to the COVID-19 pandemic, a public health emergency, health education for pregnant women has been greatly affected. On the one hand, in order to avoid crowds gathering, face-to-face antenatal classes for pregnant women cannot be carried out normally in their conventional mode; on the other hand, pregnant women, as a vulnerable group, have concerns about going out to receive conventional maternal health care services, and the amount of health education obtained directly from obstetric doctors has been reduced. In this situation, a new model of pregnancy health education is urgently needed.

**Fig. 1:**
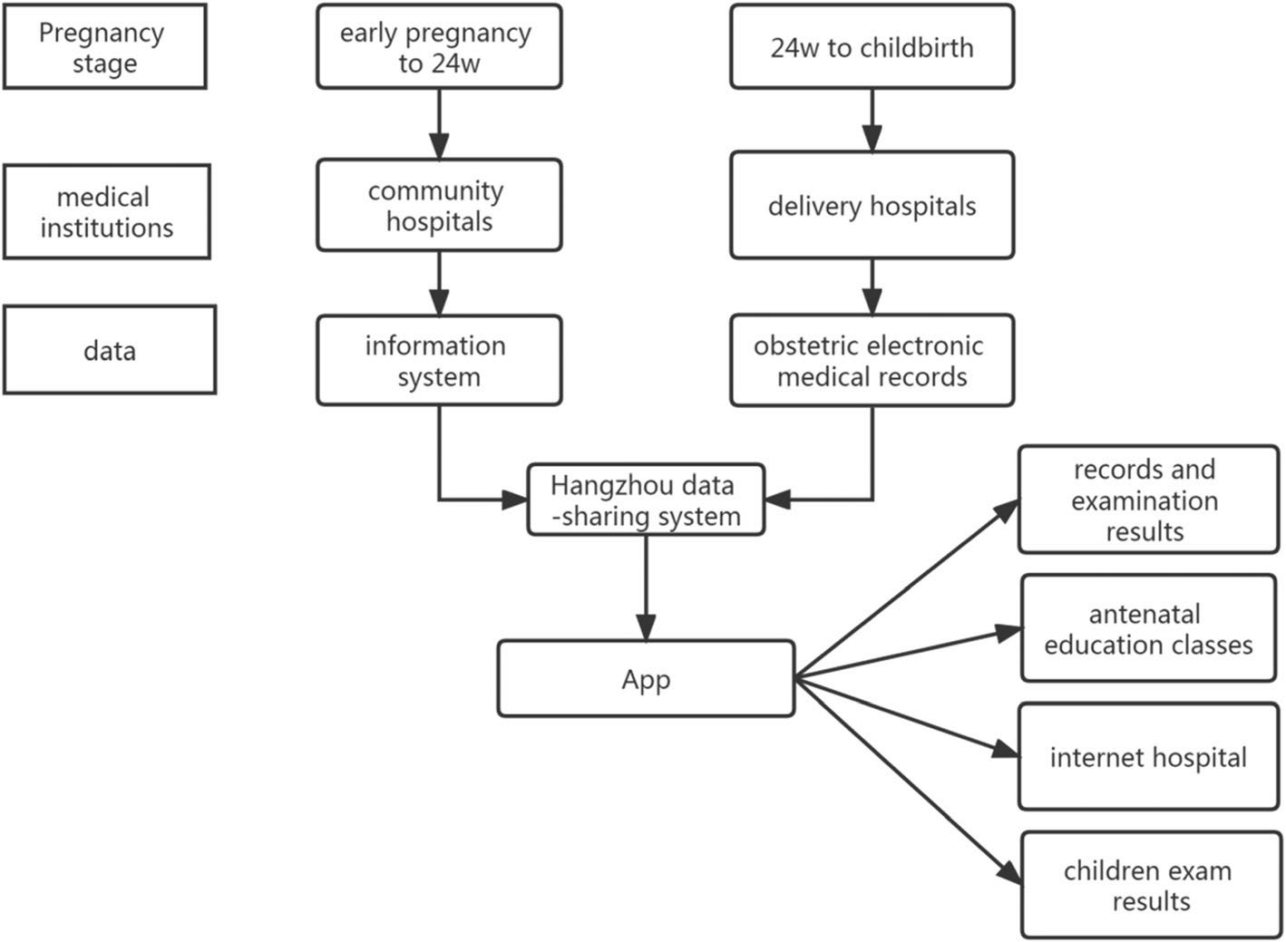
Flowchart of Pregnancy Management in Hangzhou

The management model of pregnant women in Hangzhou relies on the electronic Mother and Child Health Handbook (Fig. 1). The electronic Mother and Child Health Handbook was developed and continuously improved by Hangzhou Women’s Hospital with the strong support of relevant government departments and in collaboration with professional information institutions. It was fully promoted for use in the Hangzhou area in July 2017 [1]. Pregnant women in the Hangzhou area establish their files in community hospitals during early pregnancy and register in the Mother and Child Health Handbook app simultaneously. Pregnant women have regular prenatal checkups at community hospitals until week 24 of their pregnancy, as well as regular maternity checkups at delivery hospitals until delivery after 24 weeks [2]. Each community hospital adopts a unified information system, and each delivery hospital adopts obstetric electronic medical records, sharing the information of each maternity checkup and delivery in community hospitals and delivery institutions to the same Hangzhou data platform, which shares the data with the Mother and Child Health Handbook app. The Maternal and Child Health Handbook app is mainly composed of modules, such as records and examination results, antenatal education classes, children’s exam results, and an Internet hospital. Users can view the records and examination results of each maternity checkup, click on the learning content that matches their needs and preferences, and communicate with doctors online through the app.

## Materials and methods

### The “antenatal education classes” module of the online mother and child health handbook app in the Hangzhou Region

**Fig. 2:**
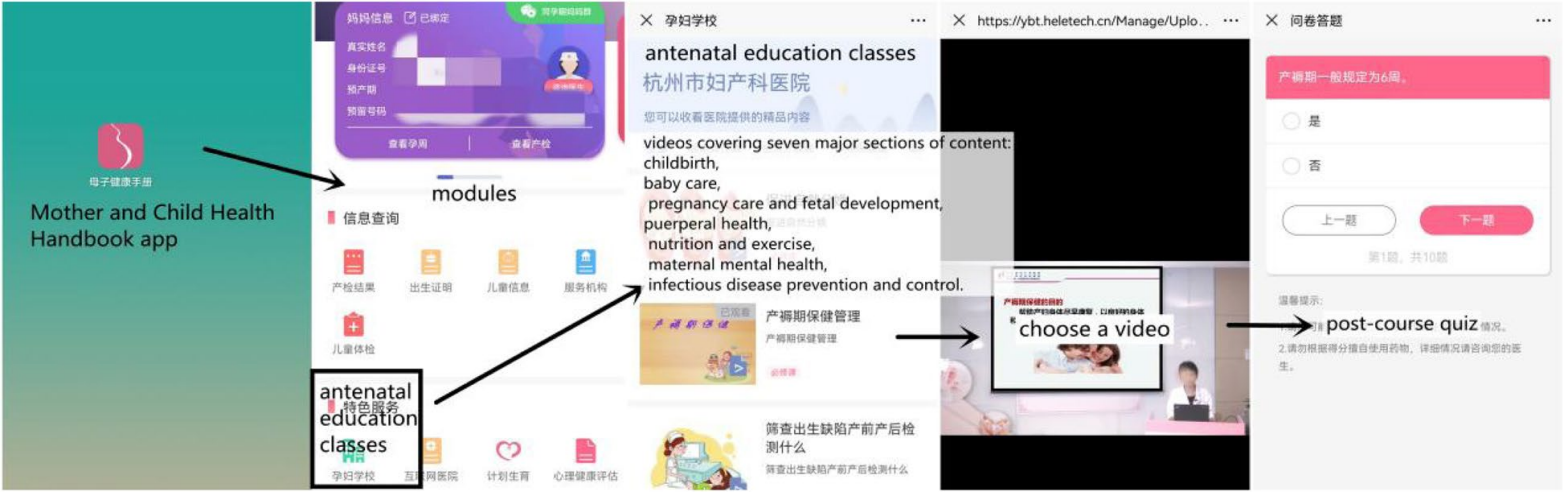
“Antenatal Education Classes” module in the app

The “Antenatal Education Classes” module uses the Mother and Child Health Handbook app as a carrier and sets up educational videos related to maternal health, covering seven topics: childbirth, baby care, pregnancy care and fetal development, puerperal health, nutrition and exercise, maternal mental health, and infectious disease prevention and control. Among them, infectious disease prevention and control is new content that was added in 2020. All course lecturers are experts in the relevant fields. Each course contains a self-administered post-course quiz, and pregnant women will take the quiz after studying each video and submit their quiz answers to receive a grade [3], as shown in Fig. 2.

### Study participants

The participants were pregnant women who established their records and registered for the Mother and Child Health Handbook app at various community hospitals in Hangzhou between January 1, 2019 and December 31, 2020. Information on the age and place of residence of these pregnant women was collected. They were divided into two groups, urban and rural, according to their place of residence; they were also divided into three groups according to age: < 18 years old (underage), 18–34 years old, and ≥ 35 years old (advanced age). The study dates, study contents, study times (watching a video and completing the corresponding online quiz were recorded as one unit), and post-class test scores of pregnant women participating in online learning were collected. All pregnant women were divided into two groups according to the time period when their profile was created: the “traditional period” group from January 1, 2019 to December 31, 2019, and the “COVID-19 period” group from January 1, 2020 to December 31, 2020. The use of online antenatal education during the corresponding time period was compared and analyzed between the two groups of pregnant women.The total amount of learning refers to the sum of the number of clicks and views of all videos within a year.The number of studies per month refers to the sum of the number of clicks and views of all videos within a month. The frequency of online studies refers to the number of times a single pregnant woman watches a video online. According to the frequency, it is divided into five groups: 1 time, 2 times, 3–9 times, 10–16 times, and more than or equal to 16 times. We compare the proportion of different groups between 2019 and 2020.

### Statistical methods

SPSS 26.0 software was used for statistical analysis, and the continuous variable data in the present study did not conform to a normal distribution and were, thus, described by median and interquartile spacing [M(QR)]. The Mann-Wilcoxon rank sum test was used for comparisons between groups. Categorical data were described as counts and percentages, and the χ2 test was used for comparison between groups for categorical variables. Differences were considered to be statistically significant at *P* < 0.05. Two-by-two comparisons between multiple groups were corrected for p-values using the Bonferroni adjustment method. All methods were performed in accordance with the relevant guidelines and regulations.

## Results

### Number and frequency of pregnant women participating in online antenatal education

**Fig. 3:**
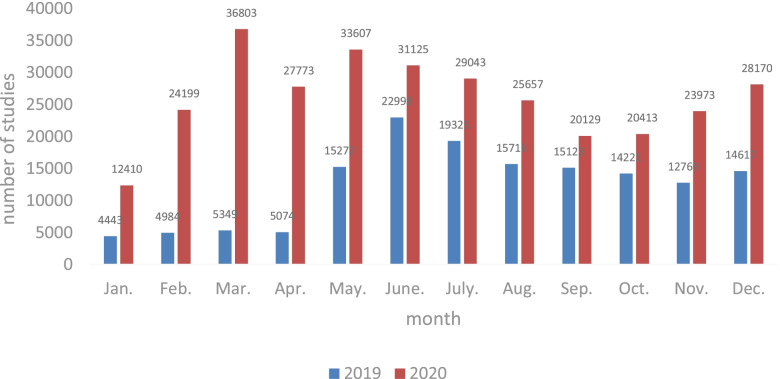
Number of antenatal education studies in the app per month

**Fig. 4:**
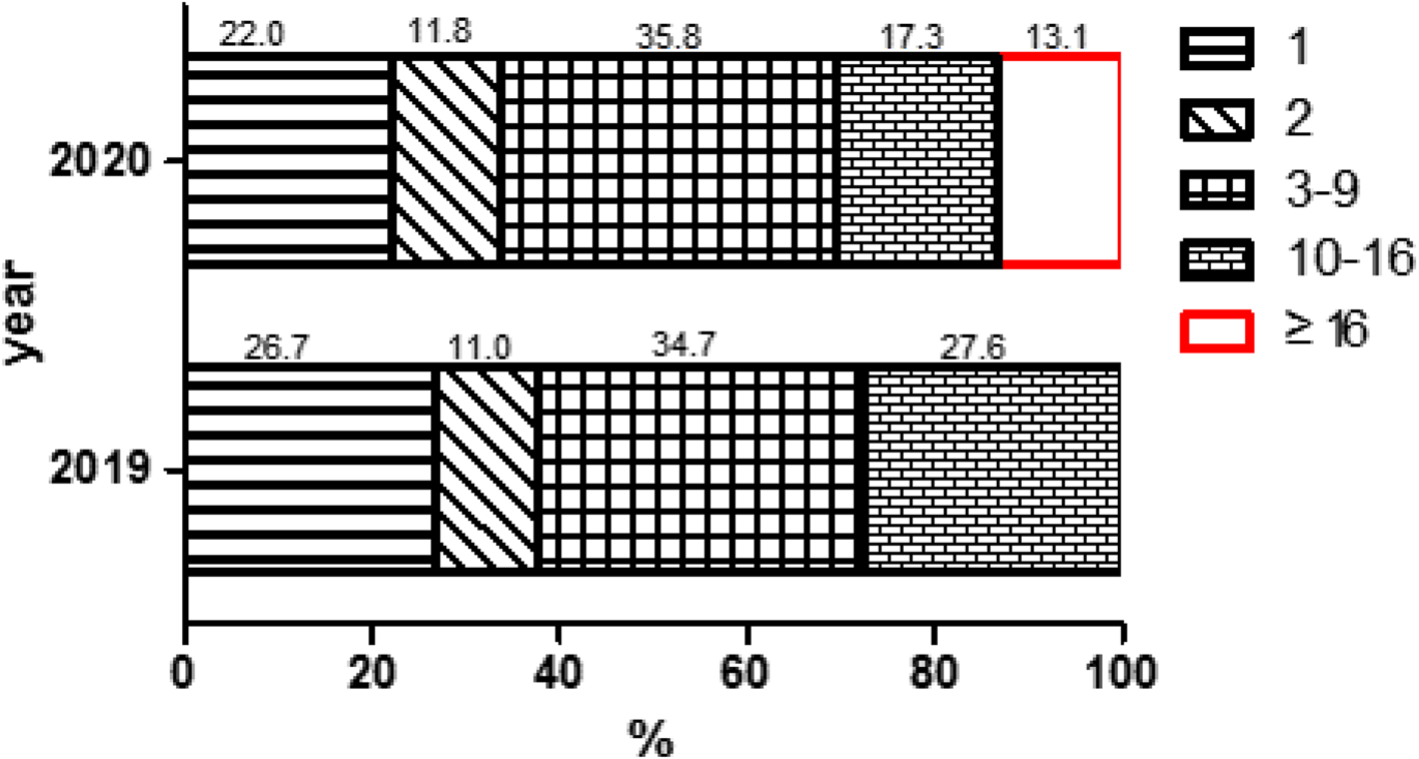
The proportion of different study frequencies

Between January 1, 2019 and December 31, 2020, a total of 229,794 pregnant women created files and registered for the app at each community health service center in Hangzhou, including 124,273 in 2019 and 105,521 in 2020; 29,226 (23.5%) participated in online antenatal education in 2019, and 36,379 (34.5%) in 2020. The difference between the participation rate in 2019 and 2020 was statistically significant (χ2 = 1857.553, *p* = 0.000). The total amount of learning was 149,891 times in 2019 and 313,302 times in 2020. The number of studies per month is shown in Fig. 3. Among pregnant women who participated in online studies in 2019, the minimum frequency of online studies for a single pregnant woman was 1, and the maximum was 16, M (QR) 4 (9) times; the minimum frequency of studies for a single pregnant woman in 2020 was 1, and the maximum was 130, M (QR) 4 (8) times. The proportion of different study frequencies is shown in Fig. 4. The results of the Mann–Whitney U test showed that differences in the distribution of learning frequencies in 2019 and 2020 were statistically significant (Z = 22.047, *p* = 0.000).

### Participation of pregnant women in learning by region and age

The minimum age of pregnant women participating in online learning in 2019 was 15 years old, and the maximum age was 52 years old, with M(QR) 29(5) years. The minimum age of pregnant women participating in online learning in 2020 was 16 years, the maximum age was 51 years, and M(QR) 29(5) years. In 2019, there were 132 pregnant women in the underage group(< 18 years old), including 34 (25.8%) who participated in online learning, 106,829 pregnant women in the 18–34-year-old group, including 25,938 (24.3%) who participated in online learning, and 17,312 pregnant women in the advanced age group (≥ 35 years old), which includes 3,253 people (18.8%), also participated in online learning. The proportion of pregnant women in the 18–34-year-old group who participated in online learning was higher than that in the advanced age group, and the difference was statistically significant (24.3% vs. 18.8%; *p* = 0.000). There was no significant difference in the proportion of online learning between the underage group and the other two groups (< 18 vs. 18–34, *p* = 0.692; < 18 vs. ≥ 35, *p* = 0.041). In 2020, there were 115 pregnant women in the underage group, including 40 (34.8%) who participated in online learning, 89,673 pregnant women in the 18–34-year-old group, including 32,029 (35.7%) who participated in online learning, and 15,733 pregnant women in the advanced age group, including 4310 people (27.4%) who participated in online learning. The proportion of pregnant women in the 18–34-year-old group who participated in online learning was higher than that in the advanced age group, and the difference was statistically significant (35.7% vs. 27.4%; *p* = 0.000). There was no significant difference in the proportion of online learning between the underage group and the other two groups (< 18 vs. 18–34; *p* = 0.834; < 18 vs. ≥ 35, *p* = 0.077). In 2019, there were 42,641 urban pregnant women, of which 15,826 (37.1%) participated in online learning, and 81,632 rural pregnant women, of which 13,399 (16.4%) participated in online learning. The proportion of urban pregnant women participating in online learning was higher than that of rural pregnant women, and the difference was statistically significant (*p* = 0.000). In 2020, there were 34,907 urban pregnant women, of which 15,564 (44.5%) participated in online learning, and 70,614 rural pregnant women, of which 20,815 (29.5%) participated in online learning. The proportion of urban pregnant women participating in online learning was higher than that of rural pregnant women, and the difference was statistically significant (*p* = 0.000).The learning profiles of pregnant women in different age groups and regions are shown in Table 1.

**Table 1:** Study of pregnant women by age group and region in 2019 and 2020

	2019					2020				
	total n	Participate in online learning		χ^2^	p	total n	Participate in online learning		χ^2^	p
Pregnancy	124,273	29,225 (23.5)				105,521	36,379(34.5)		1857.533	0
Maternal age^a^										
< 18 years old	132	34 (25.8)	< 18vs18-34	0.157	0.692	115	40 (34.8)	< 18vs18-34	0.044	0.834
18–34 years old	106,829	25,938 (24.3)	18-34vs ≥ 35	249.616	0	89,673	32,029 (35.7)	18-34vs > 35	410.435	0
≥ 35 years old	17,312	3253 (18.8)	< 18vs ≥ 35	4.158	0.041	15,733	4310 (27.4)	< 18vs > 35	3.129	0.077
Place of residence										
Urban	42,641	15,826 (37.1)		6673.187	0	34,907	15,564 (44.5)		2360.87	0
Rural	81,632	13,399 (16.4)				70,614	20,815 (29.5)			

^a^Pairwise comparisons between age groups were made three times in total. According to the Bonferroni correction method, The differences between groups compared is statistically significant at the 0.017 level(0.05/3 = 0.017)

### Participation of pregnant women in online antenatal education by different trimesters

More pregnant women accessed the online antenatal education during early pregnancy (*n* = 13,463/37.0% vs. 9088/31.1%; *p* = 0.000) in 2020 than in 2019. Similar percentages of pregnant women participated in online antenatal education during mid-pregnancy (*n* = 15,426/52.8% vs. 19,269/53.0%; *p* = 0.639) in 2019 and 2020. Less pregnant women accessed the online antenatal education during their late pregnancy (*n* = 10,246/28.2% vs. 9476/32.4%; *p* = 0.000) in 2020 than in 2019. (Table 2).

**Table 2:** Participation in online learning at different stages of pregnancy

	2019 (*n* = 29,226(%))	2020 (*n* = 36,379(%))	× ^2^	*p*
early pregnancy	9088 (31.1)	13,463 (37.0)	251.004	0.000
mid-pregnancy	15,426 (52.8)	19,269 (53.0)	0.220	0.639
late pregnancy	9476 (32.4)	10,246 (28.2)	139.862	0.000

**Fig. 5:**
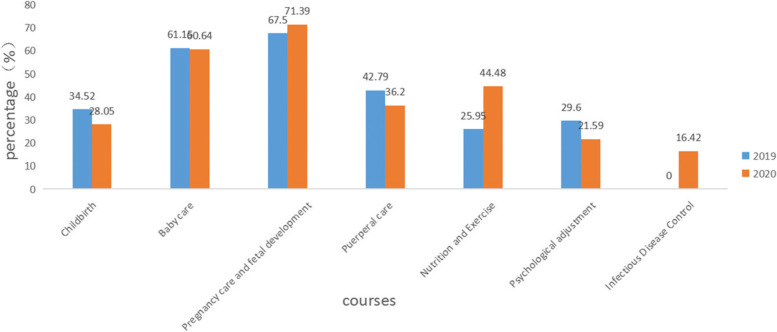
Proportion of people taking courses in the first trimester

The courses chosen by pregnant women in the first trimester are shown in Table 3 and Fig. 5. The proportion of pregnant women choosing the “Childbirth” (28.05% vs. 34.52%), “Puerperal Care” (36.20% vs. 42.79%), and “Psychological Adjustment” (21.59% vs. 29.60%) courses in the first trimester in 2020 is lower than that in 2019, and the proportion of “Pregnancy Care and Fetal Development” (71.39% vs. 67.50%), “Nutrition and Exercise” (44.48% vs. 25.95%) courses is higher in 2020 than in 2019, and there is no statistical difference in the proportion of women choosing “Baby Care” courses between the two years. The courses with the highest percentage of pregnant women in the first trimester were “Pregnancy Care and Fetal Development.”

**Table 3:** Courses chosen by pregnant women in the first trimester

Course category	2019 (n(%))	2020 (n(%))	χ2	p
	Total *n* = 9088	Total *n* = 13,463		
Childbirth	3137 (34.52)	3777 (28.05)	106.613	0.000
Baby care	5557 (61.15)	8164 (60.64)	0.584	0.453
Pregnancy care and fetal development	6134 (67.50)	9611 (71.39)	39.015	0.000
Puerperal care	3889 (42.79)	4874 (36.20)	99.166	0.000
Nutrition and Exercise	2358 (25.95)	5989 (44.48)	799.812	0.000
Psychological adjustment	2690 (29.60)	2907 (21.59)	186.416	0.000
Infectious Disease Control	–	2211 (16.42)		

*n* Number of participants in online learning. %, As percentage of total n

**Fig. 6:**
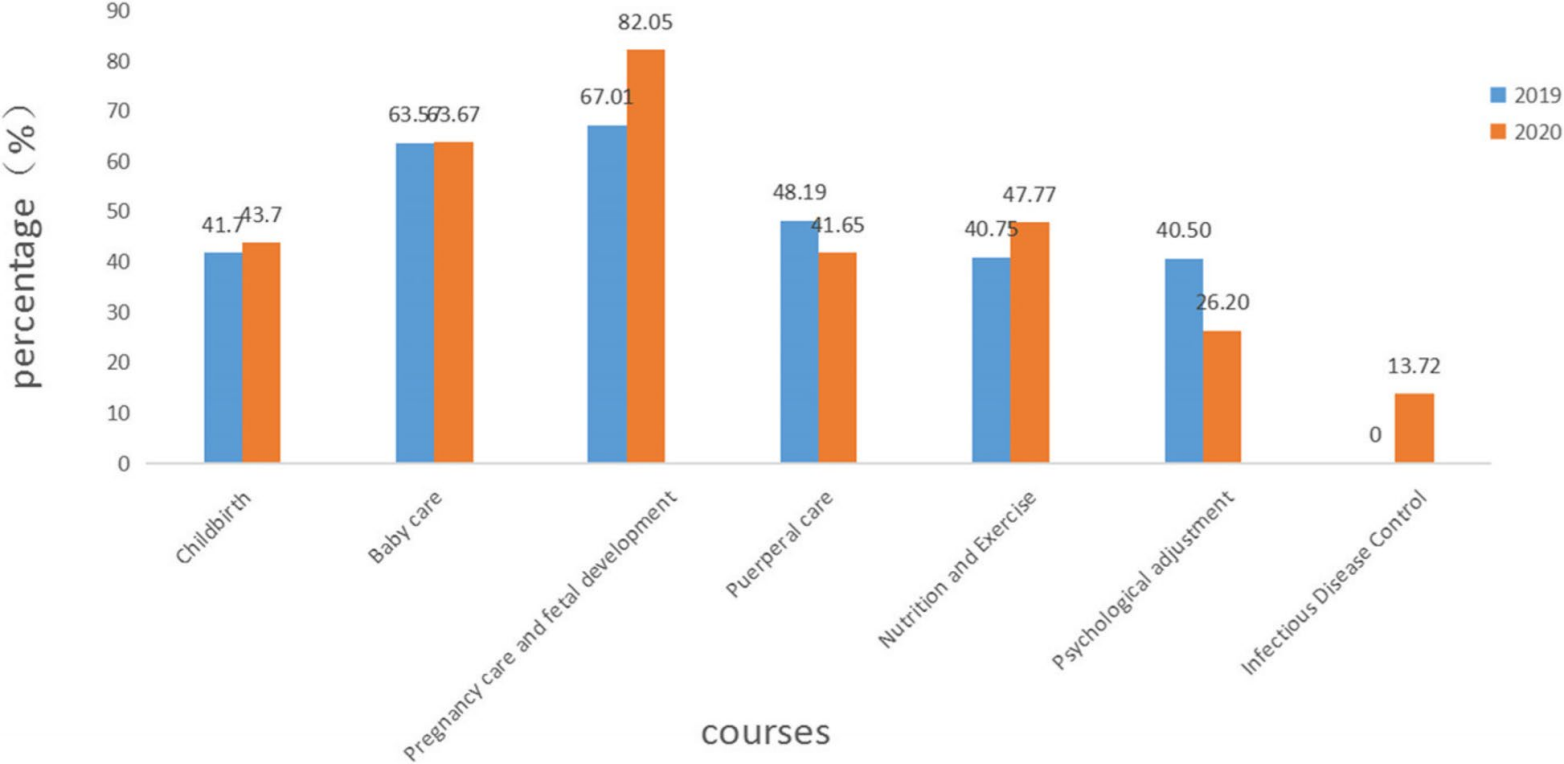
Proportion of people taking courses in the second trimester

The courses chosen by pregnant women in the second trimester are shown in Table 4 and Fig. 6. The proportion of pregnant women choosing “Puerperal Care” (41.65% vs. 48.19%) and “Psychological Adjustment” (26.20% vs. 40.50%) courses in the second trimester in 2020 is lower than that observed in 2019, and the proportion of women choosing “Childbirth (43.70% vs. 41.70%), “Pregnancy Care and Fetal Development” (82.05% vs. 67.01%), “Nutrition and Exercise” (47.77% vs. 40.75%) courses is higher than in 2019, and there is no statistically significant differences in the proportion of “Baby Care” courses between the two years. The courses with the highest percentage of pregnant women in the second trimester were “Pregnancy Care and Fetal Development.”

**Table 4:** Courses chosen by pregnant women in the second trimester

Course category	2019 (n(%))	2020 (n(%))	χ2	p
	Total *n* = 15,426	Total *n* = 19,269		
Childbirth	6432 (41.70)	8421 (43.70)	14.088	0.000
Baby care	9806 (63.57)	12,269 (63.67)	0.04	0.849
Pregnancy care and fetal development	10,337 (67.01)	15,811 (82.05)	1044.346	0.000
Puerperal care	7434 (48.19)	8026 (41.65)	148.285	0.000
Nutrition and Exercise	6286 (40.75)	9204 (47.77)	170.667	0.000
Psychological adjustment	6248 (40.50)	5049 (26.20)	797.866	0.000
Infectious Disease Control	–	2644 (13.72)	–	–

*n* Number of participants in online learning. %, As percentage of total n

**Fig. 7:**
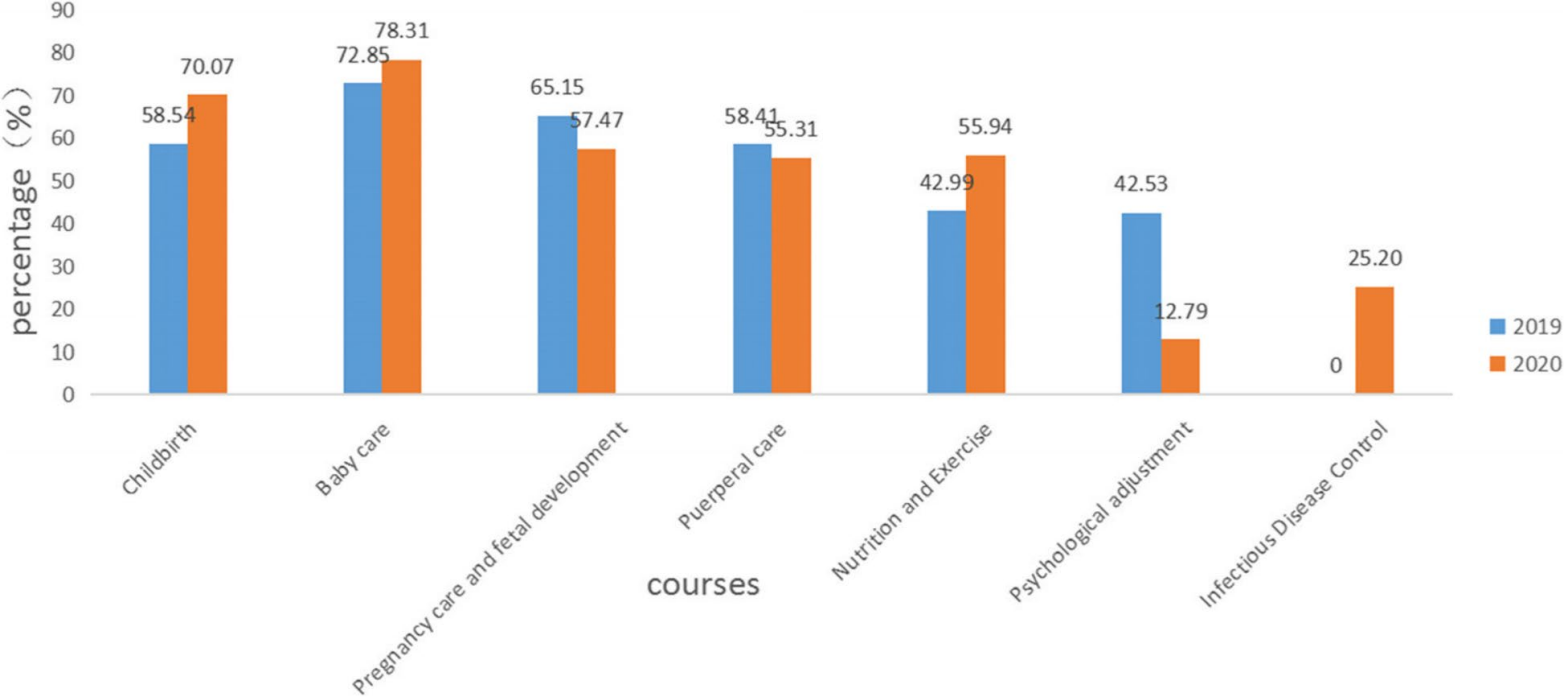
Proportion of people taking courses in the third trimester

The courses chosen by pregnant women in the third trimester are shown in Table 5 and Fig. 7. In 2020, the proportion of pregnant women learning about “Psychological Adjustment” (12.79% vs. 42.53%), “Pregnancy Care and Fetal Development” (57.47% vs. 65.15%), and “Puerperal Care” (55.31% vs. 5 8.41%) using online courses in the third trimester of pregnancy was lower than that of pregnant women in the third trimester in 2019, and the proportion of those taking “Childbirth (70.07% vs. 58.54%), “Nutrition and Exercise” (55.94% vs. 42.99%), and “Baby Care” (78.31% vs. 72.85%) courses was higher than that in 2019. The courses with the highest percentage of pregnant women enrolled in the third trimester were “Baby Care.”

**Table 5:** Courses chosen by pregnant women in the third trimester

Course category	2019 (n(%))	2020 (n(%))	χ2	p
	Total *n* = 9476	Total *n* = 10,246		
Childbirth	5547 (58.54)	7179 (70.07)	285.873	0.000
Baby care	6903 (72.85)	8024 (78.31)	79.938	0.000
Pregnancy care and fetal development	6174 (65.15)	5888 (57.47)	122.484	0.000
Puerperal care	5535 (58.41)	5667 (55.31)	19.297	0.000
Nutrition and Exercise	4074 (42.99)	5732 (55.94)	330.298	0.000
Psychological adjustment	4030 (42.53)	1310 (12.79)	2205.666	0.000
Infectious Disease Control	–	2582 (25.20)	–	–

*n* Number of participants in online learning. %, As percentage of total n

## Discussion

The COVID-19 outbreak poses a global threat to maternal health. Evidence from around the world suggests that maternal pregnancy stress is elevated [4], rates of depressive symptoms are elevated [5] [6], and many pregnant women postpone or cancel required prenatal visits [7] [8] in the aftermath of this major public health event. One study surveyed pregnant women in selected Chinese provinces and cities and showed that maternal demand for access to health information from professional maternal health care providers was very high during an epidemic emergency [9]. In a survey study of maternal health care needs of pregnant women during the COVID-19 epidemic conducted in Shanghai, China, 45.3% of pregnant women wanted to study prenatal education courses online [10]. Several studies have suggested that web-based health education can improve maternal self-efficacy [11] [12], reduce stress during pregnancy, and reduce maternal anxiety about pregnancy and childbirth [13] [14]. Online prenatal education is a means for optimizing maternal health education in the context of reducing COVID-19 transmission, especially in terms of providing pregnant women with an opportunity to learn about maternal self-care when in isolation or at home, but the actual application and effectiveness have not been widely investigated. Because many sources of information on the web are not professional medical institutions or physicians, many Chinese pregnant women are concerned about the reliability of online pregnancy knowledge, and the majority of study participants in a study of smartphone app-only use in China wanted to have web links between pregnancy apps and hospital information systems [15]. In the online antenatal education used in this study, the information system of the Hangzhou Health Administration Department was used for the management end, the app was developed by the joint information agency of professional obstetric hospitals, and the video lectures were created by experts in the corresponding fields at each hospital and were constantly reviewed and updated to ensure the reliability and timeliness of the content. During the spread of COVID-19, more courses were added to the original curriculum, and education about infectious disease prevention and control was added. This study analyzed the specific use of this online antenatal education before and after the COVID-19 outbreak, hoping to provide suggestions for a new model of health education for pregnant women during public health emergencies.

Our data suggested that the proportion of pregnant women using the online antenatal education in the app was higher in 2020 (34.5%), the special period of the COVID-19 outbreak than during the “traditional” period of 2019 (23.5%). The average single-person study frequency increased from 5.12 to 8.61. The monthly online antenatal education usage frequency was higher in 2020 than in 2019. The increase from January to April was particularly significant, which coincided with the stricter prevention and control policy in effect in Hangzhou from January to April 2020. These data indicate an increased awareness of health self-management among pregnant women and an increased demand for Internet-based health education in the special situation of reduced opportunities for offline prenatal education.

Understanding the health education needs of pregnant women plays an important role in the design and optimization of health education programs. We hope to understand the needs of pregnant women through practical applications. This study showed that “Pregnancy Care and Fetal Development” were the most selected courses by pregnant women in early and middle pregnancy, and “Babycare” was the most selected course by pregnant women in late pregnancy. This choice was not influenced by the COVID-19 pandemic, which is related to the fact that pregnant women mainly focus their emotions and attention on the fetus and baby during the perinatal period. Even at this particular time, pregnant women are more concerned about the health of others, especially their child, and to a lesser extent, the health of the fetus than their own health [16]. The proportion of childbirth course learning was higher during late pregnancy than during early and middle pregnancy, and this trend did not change before and after the COVID-19 outbreak. The proportion of pregnant women taking “Childbirth” courses during during their middle and late pregnancy was higher during the COVID-19 pandemic, considering the possibility that, in this particular case, due to fewer visits to the doctor, less information was available from the doctor, so that fear and stress about childbirth led to increased demand as the date of delivery approached. The higher percentage of depression and anxiety among pregnant women after the COVID-19 outbreak [4, 5, 6] suggests that maternal education in psychological and emotional regulation is more needed at this stage, but our data show a lower percentage of psychological courses being selected by participants (12.79%-26.20%), even lower than before the pandemic (29.6%–42.53%). This observation was unexpected, and the reasons cannot be explained at this time. Perhaps psychological teaching videos are not attractive for pregnant women to learn in this special time, and more pregnant women choose other ways to regulate their emotions. This is a speculation that requires further investigation and optimization of the model of online mental health education. The proportion of pregnant women taking nutrition and exercise courses increased during the COVID-19 pandemic, suggesting that, in this particular context, pregnant women pay more attention to nutrition and exercise during pregnancy, which is similar to the results of a Chinese survey on the demand for maternal health services during the COVID-19 outbreak [10]. During this special period, knowledge related to infectious disease prevention and control was added to our online antenatal education, but the proportion was not high (13.72%–25.20%), which may be related to the relatively low infection rate and strict prevention and control strategies in Hangzhou.

A Chinese study [17] on the use of antenatal care smartphone apps (acAPPs) by pregnant women found that higher usage of acAPPs was associated with urban residency, and the proportion of pregnant women under 35 years old using acAPPs is higher than that of pregnant women ≥ 35 years old. A Romanian study [18] on the influencing factors of pregnant women attending prenatal education lectures also found that pregnant women in urban areas are more interested in the courses. These results are similar to our study. This suggests that urban women are more receptive to this mode of health education than rural women are and that older pregnant women are less receptive to this mode of health education. online antenatal education would need to be better designed to reach rural and older women. However, due to the large amount of data, we did not account for other demographic factors or maternal pregnancy comorbidities and complications at this time, and the specific factors that lead pregnant women to choose our online antenatal education need to be further analyzed.

Due to the COVID-19 outbreak, the need for people to use the Internet as a source of healthcare information and support has increased [19]. Health education in the form of a mobile, Internet-based app can break through the time and space constraints and enable personalized learning for pregnant women in the event of a public health emergency. Based on the use of the online antenatal education, we can make preliminary speculations about the demand for maternal healthcare knowledge among pregnant women during special periods, but further research is needed for a more accurate demand survey. At the same time, the antenatal education can be optimized by combining the demand survey and data on actual usage.

## Conclusion

This study analyzed the actual use of an app-based online antenatal education before and after the COVID-19 pandemic. During the COVID-19 pandemic online antenatal education is well used by pregnant women,with “Pregnancy Care and Fetal Development” courses being the most studied by pregnant women in the early and mid-term and “Baby Care” courses being the most studied during late pregnancy. The attention to nutrition and exercise courses increased compared to the “traditional” (pre-COVID-19) period, while the proportion for psychological courses decreased. It is necessary to further optimize online antenatal education according to the actual use situation and provide convenient and personalized learning options for pregnant women during the COVID-19 pandemic, as different pregnant women have different needs for learning courses.

## Data Availability

The data will not be publicly shared to protect the participants’anonymity. The anonymized data used for analysis can be made available upon request to the corresponding author Xu-Hong Zhu.
